# Determinants of Gambling Disorders in Elderly People—A Systematic Review

**DOI:** 10.3389/fpsyt.2019.00837

**Published:** 2019-11-25

**Authors:** Morgane Guillou Landreat, Jennyfer Cholet, Marie Grall Bronnec, Sophie Lalande, Jean Yves Le Reste

**Affiliations:** ^1^EA 7479 SPURBO, Department of Addiction Disorders, Université de Bretagne Occidentale, Brest, France; ^2^Addictive Disorders Unit, University Hospital of Nantes, Nantes, France; ^3^EA 4275, Faculté de Médecine de Nantes, Nantes, France; ^4^EA 7479, Department of Family Practice, Université de Bretagne Occidentale, Brest, France; ^5^ERCR SPURBO, Department of Family Practice, Université de Bretagne Occidentale, Brest, France

**Keywords:** elderly, aged, problem gambling, pathological gambling, gambling disorder, behavioral addictions, public health, review article

## Abstract

**Background:** Despite the growth in the number of studies on gambling disorders (GDs) and the potentially severe harm it may cause, problem gambling in older adults is rarely apparent in literature. Driven by the need to overcome this limitation, a broad systematic review is essential to cover the studies that have already assessed the determinants of GD in the elderly.

**Objectives:** The aim of this systematic review is to understand the determinants related to GDs in elderly people.

**Methods:** A total of 51 studies met the inclusion criteria, and data were synthesized.

**Results:** Three major types of determinants were identified in this review: individual, socio-financial and environmental.

**Conclusions:** This review explored the determinants influencing GDs in older people. The findings are relevant to academics, policymakers, patients, and practitioners interested in the identification and prevention of GD in older people.

## Introduction

### Rationale

Gambling is a popular activity among older people and this is cross-cultural (1). Gambling participation is increasing significantly among the elderly and it is becoming a particularly widespread and regular recreational behavior among this population (2). In the United States, the participation rate in gambling among older people, over the preceding year, increased from 23% to 50%, between 1975 and 1998 (3, 4). Older adults generally possess positive attitudes towards gambling activities (5). Gambling is considered a harmless form of entertainment, whereas it was considered a vice in the first part of the 20th century (6). The rates for older adults’ participation in gambling, in the preceding year, ranged from 26.6% to 85.6% (7–10). The prevalence of problem gambling among older people ranges from 0.3% to 10.4% in studies of those over 55 years of age (3). Among those over 60 years of age, Subramaniam et al. found a life-long prevalence of problem gambling of between 0.01% and 10.6% in a systematic review (1).

In literature, a great deal of research has focused on a younger age group and on the « classic » problem gambler, especially the middle-aged man (2, 11). A broad range of risk factors for GD in young adults has been documented, including sociodemographic characteristics (male gender, younger age, low socioeconomic status) (12, 13), gambling habits (early exposure, availability) (14) or individual vulnerabilities such as negative life events, personal psychiatric/addictive comorbidity (15), or familial history of GD or substance use disorders (16, 17). More specifically, many studies on GD in adults investigated cognitive distortions which are related to an inability to control or to stop gambling (18, 19). Craving is an urge to participate in gambling and decreased cognitive control was identified in GD which correlated in fMRI with impaired activity in the prefrontal cortex (20). Reward system dysfunction was also identified in adults with GD, compared with a control group, with striatal presynaptic dysfunction (21). This point is interesting in that Dreher at al. directly demonstrated a tight coupling of midbrain dopamine synthesis and reward-related prefrontal cortex activity; they provided direct evidence for an alteration of this regulatory relationship in healthy older humans (22).

Vulnerable populations, such as elderly people, remain rare subjects in literature. However, regular gambling habits may cause potentially very serious harm: financial, social, familial, and other problems, even suicide (11, 23). Previous reviews on gambling disorders (GDs) have not focused exclusively on older people, and most of the studies had been conducted in North America: United States or Canada. Very few had been conducted in Europe.

Focusing on GDs in older adults is important, especially in order to characterise GD specificities in older adults. Several authors agree that the associated harm within this age group requires special attention (1).

### Objective

In summary, this systematic review aims to provide a broad, cross-cultural picture of the determinants of GDs in older adults. Accordingly, we reviewed both qualitative and quantitative studies that included older patients with GD.

## Methods and Materials

### Protocol, Registration, and Eligibility Criteria

The current systematic review focuses on elderly individuals with GDs and is based on qualitative and quantitative studies that describe clinical particularities. The PRISMA statement for reporting systematic reviews was adopted. The protocol had not previously been registered for this review. Inclusion criteria were coded by both authors reaching an agreement regarding the coding process and were: (a) including clinical samples of GD in those aged 65+; (b) containing quantitative and/or qualitative data; (c) being published in a peer-reviewed journal; (d) being available as a full text in one of the following languages (spoken languages of the authors): English or French.

### Information Sources and Search Strategy

Existing papers were identified by searching the academic databases PubMed and PsycINFO, from March to May 2018, published from January 1990 to February 2018. Both authors drew up a list of agreed English keywords for the systematic search: Gambling (MeSH term) OR “Gambling disorder” OR “problem gambling” OR “Pathological gambling” AND “Aged (MeSH term)” OR Aged 80+ (MeSH term) “elders” OR “older adults” in the title, abstract or keywords. Inclusion and exclusion criteria are presented in **Table 1**.

**Table 1:** Inclusion and exclusion criteria.

Inclusion criteria	Exclusion criteria
*Population*	
older people over 50 years of age	no participants over 65 years of age
gambling or problem gambling	
*Study design*	
published qualitative or quantitative studies or case reports	Websites, blogs, anecdotal evidence
*Countries, date, language*	
January 1990–February 218 studies reported in English or French	other languages

### Study Selection and Data Collection Process

**Figure 1:**
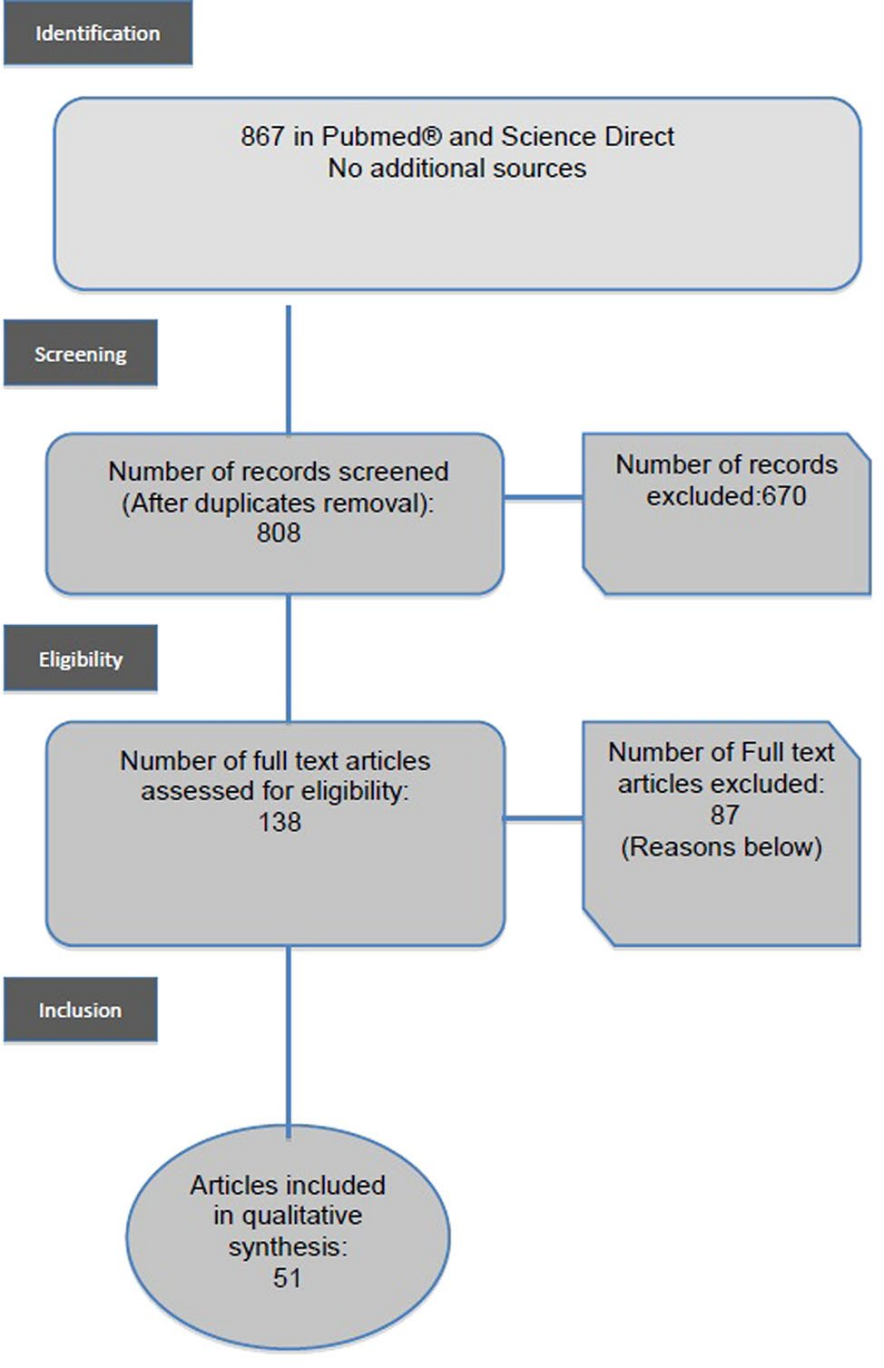
Prisma diagram.

At the first stage, 867 articles were identified with the key words Gambling (MeSH term) OR “Gambling disorder” OR “problem gambling” OR “Pathological gambling” AND “Aged (MeSH term)” OR Aged 80+ (MeSH term) “elders” OR “older adults”. At the second stage, duplicated papers were excluded. The selection of papers for the systematic review was based on the inclusion and exclusion criteria previously described. Following the search strategy presented in the flow diagram in **Figure 1**, the inspection of article titles and abstracts concluded with the inclusion of a total of 51 papers. We included studies or literature reviews in the English or French languages, which concerned older people (over 50 years of age) specifically, in clinical settings, and those which concerned gamblers (with or without problems). We excluded neurocognitive studies and experimental studies. (See inclusion and exclusion criteria—**Table 2**). We excluded studies on the general population, even when such studies included older people up to 85 years of age. Older gamblers represented a small part of these populations and so no specific analysis was carried out on these subgroups in general population studies.

**Table 2:** Article synthesis.

Country	Author	Year	N/Age median	Type of study	Main findings
**Literature review**					
France	Guillou Landreat M. et al	2017	55+	**Literature review**	GD: the main behavioral addiction in the elderly.
					Underestimation of GD in the elderly
					Treatment target: person’s quality of life and ability to regain control
France	Luquiens	2017		**Communication report**	GD: a public health problem, lack of diagnosis criteria
Canada	Mc Kay et al.	2005	55+	**Literature review**	Age/gender/gambling industry marketing strategies and slot machines (EGM): heightened risk for developing PG with EGMs in older women
Singapore	Subramaniam et al.	2015	60 +	**Literature review**	GD: 0.01% to 10.6% of GD
Australia	Tirachaimongkol et al.,	2010	55+	**Literature review**	3 clusters of risk factors: individual (distressing situations—refusal to seek help or impose barriers to gambling)/socio-environmental (unsupportive environment, cognitive distortions and incentives or misleading advertisements)/behavioral regulation factors (disinhibition, impaired decision making, risk taking)
Singapore	Tse et al.	2012	55+	**Literature review**	Several limitations: cultural, instrument, lack of empirical research, lack of qualitative study, lack of data on protective factors and positive outcomes, limited in terms of types of gambling studied
USA	Wick	2012	65+	**Literature review**	Gambling social activity for 80% elderly Elderly vulnerable to financial instability. Aged 65+ = 39% to 45% of casino traffic
**Qualitative study**					
Quebec	Papineau et al.		N = 14 65+	**Qualitative study**	Prevalence comparable to younger gamblers Retirement and social changes: risk factors Higher financial impact Specific therapeutic targets: cognitive/social/financial treatment, adapted to older people
UK	Pattinson et Parke	2017	65+	**Qualitative study**	Motivation factors: filling void/emotional escape/overspending
Singapore	Subramaniam et al.	2017	N = 25 60 +	**Qualitative study**	Gambling onset associated with family history of gambling. Gambling = family activity Financial damage in family and a significant motivating factor for seeking treatment.
Singapore	Subramaniam M. et al.	2017	N = 25 60+	**Qualitative study**	Gamblers described self-developed cognitive and behavioral control strategies to limit gambling to non-problematic levels-Comparable with middle-aged adults’ strategies
Singapore	Subramaniam et al.	2017	N = 25 60+	**Qualitative study**	Cognitive distortions: illusion of control, near miss, concept of luck, superstitious beliefs, entrapment, gamblers’ fallacy, chasing, belief that wins are higher than losses—role in maintenance of problem gambling
Australia	Tira et al.	2014	N = 31 55+	**Qualitative study**	3 pathways: grief pathway with unresolved losses/habit pathway with habituation/dormant pathway with pre-existing behavioral excess or impulsivity. Unresolved losses + mismanagement of life stresses = most significant predictors of late-life PG
**Case report**					
France	Sauvaget et al.	2015	N = 1 65+	**Case report**	Online Gambling underestimated in the elderly due to educational levels, shame, and medical and psychiatric disorders
**Quantitative study or mixed method**					
USA	Black	2017	N = 175 65+	**Quantitative study**	Older PG: women, divorced, lower level of education. Older gamblers more likely to have sought PG treatment.
Quebec	Boisvert et al.	2012	N = 54 65+	**Mixed method: Qualitative/quantitative study**	Gambling availability and characteristics (casino) respond to specific needs of the elderly
Australia	Botterill E. et al.	2016	N = 193 65+	**Quantitative Study**	Loneliness predictor of PG in older adults for men
USA	Burge et al.	2004	N = 52 65+	**Quantitative Study**	Gambling that begins in adolescence may be associated with an elevated severity of problems throughout life among older adult problem gamblers
USA	Christensen et al.	2004	N = 77 + 20 (qualitative interview) 50+	**Mixed method Quantitative/qualitative Study**	No correlations between gambling and health perception
New Zealand	Clarke D et al.	2008	N = 104 65+	**Quantitative study**	Gambling less severe but more frequently Gambling severity correlated with motives for releasing tensions
Canada	Cousins et al.	2007	N = 444 65+	**Quantitative study**	At risk/bingo gambling: female, living in rental accommodation, receiving federal income and reporting health problems, and also sedentary: predictors of more money spent on bingo.
USA	Desai et al.	2004	N = 2,417 65+	**Quantitative study**	Recreational Gambling in older adults not associated with negative measures of health and well-being
USA	Desai et al.	2007	N = 43,093 65+	**Quantitative study**	PG is associated with poorer health measures. Recreational gambling was associated with negative measures (obesity)+with positive measures (mental and physical functioning)
USA	Erickson et al.	2017	N = 343 60+	**Quantitative study**	6.4% problem gamblers, 1.8% pathological gamblers. physical and psychological distress in PG
Finland	Joutsa et al.	2014	N = 575 43–90	**Quantitative study**	7% PG Correlated with depression
USA	Kausch et al.	2004	N = 37 60+	**Quantitative study**	psychiatric disorder, suicidal ideation comparable to younger people
USA	Kerber et al.	2008	65+	**Quantitative study**	High level of psychiatric comorbidities
USA	Kerber at al.	2015	N = 40 65+	**Quantitative study**	Gambling causing depression, being fired from a job due to gambling, and still paying off gambling debt
USA	Ladd	2003	N = 492 65+	**Quantitative study**	Lifetime rates of PG: 12.9% in the bingo sample and 9.7% in the senior center sample. 39.1% reported gambling at least 2×/month, and 33.7% wagered >50 dollars over the prior 2 months.
Canada	Lai et al.	2006	N = 2,272 55+	**Quantitative study**	26.6% had gambled. Male, having lived in Canada longer, a higher level of social support, more service barriers, stronger level of Chinese ethnic identity associated to higher probability of gambling
USA	Levens et al.	2005	N = 843 65+	**Quantitative study**	69.6% gambled in the past year. 10.9% at risk gamblers
USA	Martin et al.	2011	N = 247 60+	**Quantitative study**	Complex intrinsic and extrinsic motives for casino venues: entertainment/win/money/allay boredom/loneliness
USA Brazil	Medeiros et al.	2015	N = 70 65+	**Quantitative study**	Significant differences between 2 cultures: gambling course, age of initiation, gambling characteristics and behavior, personal history and antecedents
USA	McNeilly et al.	2000	N = 315 65+	**Quantitative study**	main motivations for gambling: relaxation, boredom, passing time, and getting away for the day
Australia	Nower, Blaszcynski	2008	N = 1,601 56+	**Quantitative study**	Sex differences, women: telescoping effects, Preference for non-strategic games. Fear of suicide: a factor motivating self-exclusion
UK	Parke et al.	2018	N = 595 65+	**Quantitative study**	Late-life PG: escape anxiety resulting from deteriorating physical well-being/social support/induced depressive states
USA	Petry	2002	N = 49 55+	**Quantitative study**	A minority of older PGST. Gender differences: women = late age of regular gambling and wagering high amounts
Quebec	Philippe et al.	2007	N = 810 55+	**Quantitative study**	At-risk gambling: 1.6% Pathological gambling: 1.2%
USA	Pietrzak et al.	2007	N = 10,563 60 +	**Quantitative study**	Lifetime recreational gamblers: 28.7%, 0.85% Higher medical, addictive and psychiatric comorbidities in PG
USA	Pietrzak et al.	2006	N = 31 60+	**Quantitative study**	75% of pathological and 30% of problem gamblers interested in gambling treatment Problem gambling induces increased psychological distress in older adults
USA	Pilver et al.	2016	N = 10,563 55+	**Quantitative study**	Gambling positive activity for older adults but risky and PG associated with psychiatric disorders
USA	Piscitelli et al.	2017	N = 2,103 55+	**Quantitative study**	18.5% would visit a casino, stay longer, and spend more money if new casino open close to them
USA	Potenza et al.	2006	N = 1,018 55+	**Quantitative study**	Older gamblers: Lower income, lower duration of gambling, fewer types of gambling, more problems with slot machines
USA	Singh et al.	2007	N = 300 65 (mean)	**Quantitative study**	Parkinson and gambling Patients with PG younger than other patients
Australia	Southwell et al.	2008	N = 414 60+	**Quantitative study**	Predictors of PG: Younger, male, single, motivated to play EGMs (excitement/to win money) 27% reported drawing on their savings to gamble
Singapore	Tse et al.	2013	N = 3,010 55+	**Quantitative study**	39.2% gambled in the past year; O.9% had PG (2.2% of the population of lifetime gamblers). Type of gambling: continuity without set limits to amount wagered
USA	Vanderbilt J. et al.	2004	N = 1,016 70+	**Quantitative study**	47.7% reported gambling. Gambling: a form of social support. Younger age, greater social support, alcohol use in the past year associated with gambling activity
Canada	Van der Maas, et al.	2017	N = 1,978 55+	**Quantitative study**	Using bus tours to access Canadian gambling venues associated with risk of PG. Bus tours patrons likely to be: female, over 75 years old, born outside Canada
Canada	Wiebe et al.	2005	N = 1,000 60+	**Quantitative study**	74.7% gambled in the past year, 1.6% problem gambling. South Oaks Gambling Screen – R: needs to be refined for use with older adults
USA	Zaranek et al.	2005	N = 1,410 60 +	**Quantitative study**	Majority of social gamblers 17.2% visited the casino monthly or more frequently Positive attitudes about casinos
USA	Zaranek et al.	2008	N = 1,410 60+	**Quantitative study**	Problem gambling: 10.4%, 18% among those reporting casino visits

### Data Extraction and Quality Assessment

Considering the exploratory nature of this systematic review, and in order to have a broad understanding of GD in the elderly, the studies were not filtered according to their quality and both qualitative and quantitative studies were taken into consideration. Given the high levels of heterogeneity of the data across studies, as regards research methods, data were synthesized qualitatively through a summary table and a narrative synthesis using these categories: (1) the individual determinants; (2) the structural determinants; (3) the environmental determinants.

## Results

### Study Selection and Characteristics

In this review, the first group of 867 papers was identified by searching for the keyword in the scientific database. As described in the flow diagram (**Figure 1**), 670 papers were excluded because they were replicated records or because the topic was not GD in elderly people. One hundred thirty-eight were assessed for eligibility, 87 full texts were excluded because they did not describe clinically elderly persons with GD. A total of 51 studies met the inclusion criteria. The publication dates ranged from 1990 to 2018 and contained clinical samples of elderly persons with GD. Selected articles are listed in **Table 2**.

### Risk of Bias Within Studies

#### Selection Bias

The definition of older adults significantly differed in literature: 12 articles specified those over 50 or 55 years of age (3, 9, 10, 24–32); 16 articles, those over 60 years of age (1, 2, 5, 33–45); 16 articles, those over 65 years of age (32, 46–60), and 7 over 70 years of age (8). Methodologically, these differences in age criteria bring difficulties in comparing and analyzing data from the literature.

#### Evaluation Bias

Gambling becomes a problem when gambling behavior becomes persistent, recurrent, and leads to clinically significant difficulties (61). The diagnostic criteria for pathological gambling are constructed and validated for the middle-aged adult in employment. Several criteria lose their specificity in ageing subjects: loss of social activities, damage to career, and harm to those close to them, are far less relevant to retired people who are alone or isolated for reasons other than those related to gambling behavior (62).

#### Cultural Bias

A large majority were conducted in North America (USA or Canada), Australia, or Singapore but only a few articles concerned Europe (six articles).

### Synthesis of the Results

**Figure 2:**
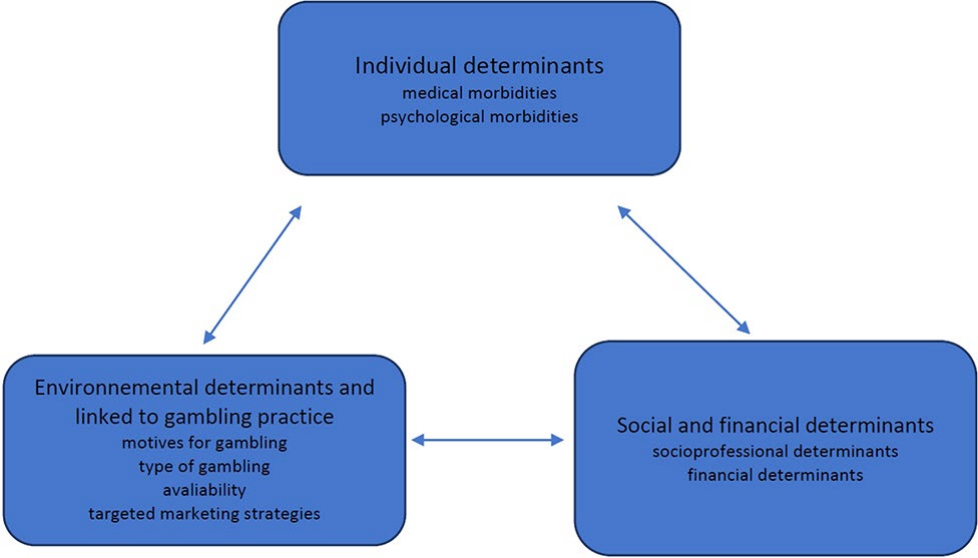
Themes identified the review.

Articles are presented in **Table 2**. 3 Themes were mainly identified (**Figure 2**)

This review focuses on: (1) individual determinants, (2) structural determinants, (3) environmental determinants of GD in the elderly.

#### Individual Determinants

Gender influences gambling habits. Women over the age of 60 have a risk of problem gambling that is equivalent to, or even higher than, that of men in the same age group (20). The prevalence of at-risk or problem gamblers (between 1 and 4 in the criteria for pathological gambling) is high among women over 65 years of age (20).

Age at gambling initiation is a risk factor for more frequent and more severe gambling behavior, as well as for pathological gambling in later life (3, 21). However, where a more advanced age is concerned, most studies found that the prevalence of problem gambling declined with age beyond 60. Ladd et al. conducted a study among gamblers over 65 years of age and found problem gamblers were significantly younger than non-problem gamblers (22).

#### Comorbidities

In elderly subjects with gambling problems, more significant medical or psychosocial comorbidities are reported than in non-gamblers or non-problem gamblers (1). These connections are multifactorial. Gambling is a sedentary activity, which can lead to medical problems (23) or, conversely, it can attract people who already have difficulty moving around for medical reasons. People with alcohol use disorder or tobacco use disorder, whose general health is impaired, may seek relaxation or excitement from readily available gambling opportunities (1). Moreover, the practice of gambling is in itself a stress factor. Several studies have shown neurophysiological changes (hyper-reactivity of the autonomous system in response to gambling-related stimuli) and neurochemical changes (elevated levels of cortisol, norepinephrine, and dopamine during casino gambling sessions). These changes may increase the risk of chronic pathologies (51).

Older adults with a life-long history of problem gambling had experienced significantly more medical problems in the previous year (1). Problem gambling is associated with chronic medical illness (24). Pietrzak et al. showed that, among medical morbidities in older adults with PG, angina and arthritis were overrepresented (25) and both reduce the physical abilities of older adults. Problem gambler status is significantly associated with a lower quality of life, in medical, social, and emotional terms, compared with social gamblers or non-gamblers. They have a more negative perception of their general and psychological state and a more pessimistic view of their future state of health (26, 27). Desai et al. (32) showed, in data from the NESARC (National Epidemiologic Survey on Alcohol and Related Conditions), that subjects over 65 years of age, with a history of problem gambling during the year, had significantly more alcohol use disorders and were more frequently tobacco dependent (24). Life-long problem gambling was associated with substance use disorders (alcohol, illicit substances), but also with psychiatric disorders: mood disorders (depression, dysthymia, mania, hypomania) and anxiety disorders or avoidant personality disorder (25, 28). In older adults, cognitive impairment may also reduce the ability to decide to stop gambling (29), especially in cognitive impairment which involves the frontal cortex (as in frontotemporal lobar degeneration).

Specific attention should be paid to the complex and varied relationship between gambling and Parkinson’s disease (PD) and dopaminergic medication (30). Impulse control disorders (ICDs), such as compulsive gambling, and also buying, sexual, and eating behaviors, are a serious, recognized complication in PD which occurs in up to 20% of PD patients over the course of their illness and especially in those with the highest risk profile (31).

#### Social and Financial Determinants

Several studies identify socio-professional, financial, and educational status as risk factors for problem gambling (1, 23). This connection is not found in all studies (22) but it should be taken into account, particularly with regard to the elderly. Older people are especially vulnerable to gambling related problems due to loss experienced in their personal life: loss of role, loneliness, social isolation, and a lower or fixed income. The change in professional status with retirement can have a direct influence on a gambler’s behavior. A decline in income in retirement can be a factor which precipitates the passage from social gambling behavior to problem gambling. Gamblers may wish to make up for a loss of income through winnings from gambling by increasing their participation (26, 37). The money-making motivation, combined with the search for excitement, have been identified as predictors of risk and problem gambling among slot-machine gamblers over the age of 60 (38). In addition, the failure to adjust their betting in proportion to their lower income in retirement may also lead to difficulties.

#### Determinants Correlated to Gambling

##### Cognitive Distortions

Cognitive distortions, found in younger adults (63), have also been identified in older adults. In a multi-ethnic Asian sample of gamblers, Subramaniam et al. identified the following themes in the perception of gambling: skill, near miss, concept of luck, superstitious beliefs, entrapment, gambler’s fallacy, chasing wins, chasing losses, and the belief that wins exceeded losses. These gambling-related cognitions played a role in the maintenance and escalation of gambling (39).

The type and structural characteristics of gambling may enhance cognitive distortions. Lottery video terminals and slot machines have used a computer and virtual reels to determine the odds. Since the end of the eighties, a clustering technique has been used to create a high number of near misses (64). What the gamblers see on the machine does not correspond to reality and it induces a misperception of the probability of winning; gamblers take the near miss as an indication of their improving skills which leads to gambling behavior being sustained (65).

It could be interesting to analyse older problem gamblers’ cognition to identify strategic prevention targets. A reduction in gambling cognitive distortions was identified as being one of the best predictors of recovery (66).

##### Motives for Gambling

Eighty percent of older gamblers are looking for entertainment and enjoyment (5, 67). Thirty-eight percent say they gamble to distract themselves from everyday problems, and combat boredom but also loneliness (32, 34, 58). Studies in North America show that older people frequently visit gambling locations (casinos) to make social connections (2). Living alone and/or being separated, divorced or socially isolated are factors associated with problem gambling (1).

Access to stimulating activities for leisure and pleasure are likely to be reduced with age and some people may not be able to participate in activities they had previously enjoyed (1, 54). One hypothesis is that gambling fills a void in the lives of older people and may be a form of substitute for social support (33).

The fight against negative emotional states (68), linked to loss or grief, is one of the factors motivating gambling activity (3, 67). However, gambling is not always a problem: improving cognitive skills is one of the motives for gambling. A general population study has even shown that people who had gambled during the past year had better subjective health than those who had not gambled (69).

##### Type of Gambling

Casino trips are the first outside activity offered to institutionalized elderly people (67). Over 65s account for 39%–45% of all casino users. In terms of casino use, the prevalence of pathological gambling ranges from 6.7% (never) to 19.1% (at least once a month) in a study conducted among non-institutionalized elderly subjects (33).

The use of gambling varies among older subjects according to the characteristics of the type of gambling practiced. The perception of harm associated with gambling products is high in the general population; casino and EGMs are identified as very, or extremely, harmful by the general population. According to TSE et al. in 2013, problem gamblers over the age of 55 tend to play continuous or limitless games such as slot machines, online games or even scratch games, while those without a gambling problem tend to play discontinuous, inexpensive and time-limited games such as lotteries. However, there are few studies so far on the structural characteristics or the different types of games (pure chance games or those involving skill) which are popular with the elderly (3).

##### The Availability of Gambling Opportunities

One of the factors highlighted by this increase in gambling behavior is the expansion of the legal gambling market, especially with developments in Internet gambling. The global gambling market was estimated to be worth 430 billion US dollars in 2012 (Global Gambling and Gaming Consultants). Several studies have highlighted the links between the availability and proximity of gambling opportunities and excessive gambling practices (8, 70). A study on socio-cultural factors among gamblers over 60 years of age showed that, in problem gambling populations in need of care, the age at initiation and the desire to gamble were much higher in the United States than in Brazil (53). One of the authors’ hypotheses is that the availability of gambling, as a result of each state’s legislation, is much more significant in the USA than in Brazil. The legislative framework for gambling can thus have a direct impact on gambling practices (53), especially among vulnerable people.

Gambling is part of a growing industry driven by powerful multi-national corporations. There is an intensification in marketing strategies which target older people (26). Older adults are an especially desirable demographic for the gambling industry because they fill the floors during off-peak hours. Casinos aggressively direct marketing towards them, offering discounts on meals, free drinks, and guarantees to win and, sometimes, medication discount coupons. Some gambling locations also offer transportation for people coming to the casino (71). A recent study showed that, among older adults, using bus tours to access gambling venues was associated with an increased risk of problem gambling (28, 60).

Loyalty strategies are being implemented by many casinos in France which offer a “VIP” upgrade to gamblers. The gambling opportunities offered at casinos are described as the ideal solution for the “needs of seniors”, and several countries are trying to raise awareness of this intrusive marketing campaign which targets vulnerable, elderly people (67, 72).

The targeted gamblers most susceptible to these offers (bus tours) tend to be retired women, over 75 years of age (28, 60). Older women seem to be more vulnerable to gambling marketing strategy and, more particularly, to electronic games machines (73). Specific gambling characteristics in older adults are synthetised in **Table 3**.

**Table 3:** Specificities of gambling characteristics in older adults.

	Older adults
**GD screen tools**	No specific tools Less specificity of criteria : occupational / social consequences
**GD lifetime prevalence**	0.01-10.6% (Subramaniam, 2016) Percentage of pathological gambling decreasing with age beyond 60
**Gambling determinantsIndividuals**	Women over 60 years old
**Social determinants**	Losses , isolation, lower and fixed income , retirement
**Motives for gambling**	Entertainment, enjoyment Combat boredom, fight against negative emotional states, fills a void Social connections, substitution for social support Improving cognitive skills
**Gambling characteristics**	Expansion of legal market, availability, accessibility Targeted, intrusive marketing strategies Type of gambling: casino, continuous and limitless games (for PG)

## Discussion

In a large majority of studies, gambling in older people is compared with gambling in a younger population in employment. Through the analysis of the selected articles, clear gambling specificities in older adults were identified: first, individual specificities; second, social and financial specificities; and third, those correlated with gambling.

Individual determinants, concerning gender and age and morbidities could make it possible for caregivers for the elderly to identify GD in this population and offer guidance. Chronic medical illness, (32, 43), substance use disorders, but also mood disorders, anxiety disorders or avoidant personality disorder (43, 55), cognitive impairment (49) or PD (49) are risk factors for GDs (74). Social and financial determinants are also specific keys to GDs in older adults. Retirement is a moment when some are at risk, particularly at risk of failure to adjust their betting in proportion to their lower income (26, 45). Disordered gambling may increase financial problems, including credit card and other debts (75). A reduced ability to deal with the damage caused by their gambling is one particular specificity in older adults. Lack of resources to cover the damaging level of gambling expenditure appears to be specific to older adults. They have less time and fewer financial resources to recover from social, financial and, particularly, the medical and psychiatric consequences resulting from disordered gambling. Therefore, the identification of GDs is not initially concerned with medical care and so other types of action could be required to help gamblers to control or to stop their habit. Family support, for example, is very important: a recent study showed that, among older gamblers, family support was essential in helping to implement control strategies for responsible gambling (40). To help identify GD in vulnerable older people, Kerber et al. proposed the acronym “CASINO” to help everyone to remember the impact and factors linked to disordered gambling in older adults: Chronic health problems, Affective disorders, Serious risk of suicide, Incarceration, NO money, credit card debts, and financial problems (57). This acronym includes individual and social determinants.

One main point of this review is that it underlines the influence of determinants correlated with gambling. Gambling type, as well as gambling-targeted offers and availability, reinforce gambling motivation and cognitive distortions in older people. An editorial in the journal, Nature, underlined recently that gambling in vulnerable populations is a public health concern. They asked: how can research help the unfortunate minority who cross to gambling’s dark side? They also drew attention to the lack of scientific studies on the subject and to the lack of debate about society taking control of an industry which profits from compulsive gambling much more than from occasional gambling (76). In 2009, Moodie and Hastings pointed out that public health authorities could learn a great deal from tobacco control, in terms of how to respond to gambling (77). Caregivers and public health authorities should be aware of specific points concerning gambling practices in older adults. Gambling can be extremely attractive and easily available to the elderly. All gambling marketing variables are adapted to fit older people’s needs and vulnerabilities and to increase gambling activity.

Casinos and other gambling locations know how to meet the specific needs of older people. Isolation and boredom are risk factors for GD in older adults. Public authorities and institutions taking care of older people should consider that to delegate the provision of social activities for older people to gambling locations, as defined in literature, may not be an ideal solution and may not demonstrate a responsible or fair attitude towards older people. Social or leisure activities could be developed which are suited to older adults and which would help to limit casino attendance and reduce harm. Gambling locations direct extensively aggressive marketing towards older people.

The extent to which these assisted living facilities should encourage older adults to gamble increasingly and whether they should be liable for increasing the financial risks of the residents are matters to be addressed. The risk is especially important if one fails to identify gambling problems in older adults.

### Limitations

Due to the lack, in current literature, of specific analysis on elderly gamblers, literature review, case reports, qualitative, and quantitative studies were included in this systematic review. It induces a high level of heterogeneity of data.

Results of this review are limited by three bias, which limits comparison of data. We identified selection bias in selected studies and cultural bias, as a majority of studies concern North America. An evaluation bias was also identified, it concerned the definition of problem gambling or GD in older people, which differed in articles. It raised problems in comparing and analyzing the existing data.

## Implications and Conclusion

A wide variety of treatment options is available for gamblers who seek help and treatment: phone lines (psychiatric emergencies or gambling helpline), associations (e.g., Gamblers Anonymous), outpatient treatment (private therapists, community mental health centers or addictive disorders centers) but also general practitioners and, sometimes, in-patient addiction recovery centers. Very few older people with a GD will seek access to specific treatment programs. Therefore, to reduce harm, especially financial, social, and psychological harm, family and social services have a principal role to play and a protective legislative measure could be discussed. However, policies concerning gambling control are still insufficient for helping vulnerable gamblers to reduce their gambling activities.

According to literature, it seems that older adults are gambling more and more and that the proportion of pathological gamblers is increasing in this age group. The findings of this current review support the need to consider the determinants of gambling in this group. Pathological gambling among older adults is associated with medical, psychiatric, and social comorbidities. The types of motivation for gambling in older adults involve the search for entertainment and the fight against boredom and loneliness. It has the potential to cause extreme harm because of a lack of resources to recover from the negative consequences of gambling. Many studies underline vulnerabilities, especially those linked to the environment and to gambling. Public health authorities and societies should take these findings into account. Gambling policies should help vulnerable gamblers to better control the habit and to reduce harm caused by gambling. There is a need to question the responsibility of public health authorities, as well as the lack of legislation and social measures to control gambling marketing strategies and gambling availability targeting vulnerable people. As with other addictions, responsible governments need to balance tax revenue against a duty of care towards vulnerable members of society (78)

## Author Contributions

MGL, JC, and MGB carried out the systematic search of bibliographic databases, and reviewed all the articles. MGL, JC, and MGB wrote the article. SL and JR reviewed and revised the article.

## Conflict of Interest

MGB and JC declare that the University Hospital of Nantes has received funding from the gambling industry (FDJ and PMU) in the form of a sponsorship that supports the gambling section of the BALANCED Unit (the Reference Center for Excessive Gambling). We guarantee that this review is scientifically independent of the gambling industry operators. There were no constraints on publishing.

The remaining authors declare that the research was conducted in the absence of any commercial or financial relationships that could be construed as a potential conflict of interest.

## Abbreviations

GD, Gambling disorder.
